# Evidence of autoinflammation as a principal mechanism of myocardial injury in SARS-CoV-2 PCR-positive medical examiner cases

**DOI:** 10.1186/s13000-023-01397-7

**Published:** 2023-10-18

**Authors:** Margo E. Hammond, Erik D. Christensen, Michael Belenky, Gregory L. Snow, Kevin Shah, M. Elizabeth H. Hammond

**Affiliations:** 1 Department of Chemistry and Biochemistry, Brigham Young University, Provo, UT USA; 2 Office of the Medical Examiner, Utah Department of Health and Human Services, Salt Lake City, Utah USA; 3 Office of Research, Intermountain Medical Center, Salt Lake City, Utah USA; 4 Cardiology Division, University of Utah School of Medicine, Salt Lake City, Utah USA

**Keywords:** SARS-COV-2, Autoinflammation, Neutrophils, Heart, Myocarditis, Complement, Coagulopathy

## Abstract

**Background:**

Disease from Severe Acute Respiratory Syndrome Coronavirus-2 (SARS-CoV-2) remains the seventh leading cause of death in the United States. Many patients infected with this virus develop later cardiovascular complications including myocardial infarctions, stroke, arrhythmia, heart failure, and sudden cardiac death (20–28%). The purpose of this study is to understand the primary mechanism of myocardial injury in patients infected with SARS-CoV-2.

**Methods:**

We investigated a consecutive cohort of 48 medical examiner cases who died with PCR-positive SARS-CoV-2 (COVpos) infection in 2020. We compared them to a consecutive cohort of 46 age- and sex-matched controls who were PCR-negative for SARS-CoV-2 (COVneg). Clinical information available at postmortem examination was reviewed on each patient. Formalin-fixed sections were examined using antibodies directed against CD42 (platelets), CD15 (myeloid cells), CD68 (monocytes), C4d, fibrin, CD34 (stem cell antigen), CD56 (natural killer cells), and myeloperoxidase (MPO) (neutrophils and neutrophil extracellular traps(NETs)). We used a Welch 2-sample T-test to determine significance. A cluster analysis of marker distribution was also done.

**Results:**

We found a significant difference between COVpos and COVneg samples for CD42, CD15, CD68, C4d, fibrin, and MPO, all of which were significant at p < 0.001. The most prominent features were neutrophils (CD15, MPO) and MPO-positive debris suggestive of NETs. A similar distribution of platelets, monocytes, fibrin and C4d was seen in COVpos cases. Clinical features were similar in COVpos and COVneg cases for age, sex, and body mass index (BMI).

**Conclusion:**

These findings suggest an autoinflammatory process is likely involved in cardiac damage during SARS-CoV-2 infection. No information about clinical cardiac disease was available.

## Introduction

Severe Acute Respiratory Syndrome Coronavirus-2 (SARS-CoV-2) caused a global pandemic. Disease from SARS-CoV-2 is currently the seventh leading cause of death in the United States [1]. Many patients infected with this virus eventually develop cardiovascular (CV) complications including myocardial infarctions, stroke, arrhythmia, heart failure, and sudden cardiac death. Specifically, patients with SARS-CoV-2 have a high prevalence of severe myocardial injury (20–28%) [2–4]. The mechanism of cardiac injury in patients dying of SARS-CoV-2 after severe respiratory illness followed by cardiovascular complications is controversial. Direct viral infection as well as various indirect causes have been postulated [2–7]. A recent study comparing 153,760 US SARS-CoV-2 infected veterans to a large cohort of normal controls showed the risk of cardiovascular outcomes hazard ratio for any prespecified cardiovascular outcomes regardless of acute presentation was 1.63 ± 0.09 [2]. We hypothesized that innate immune activation (autoinflammation) and its consequences are the primary cause of cardiovascular disease in SARS-CoV-2 patients. We used immunohistochemistry and routine histology to assess our hypothesis.

## Methods

### Population

We investigated a consecutive sample of 49 PCR-positive SARS-CoV-2 (COVpos) patients who underwent postmortem examination in 2020. This series of cases was collected prior to the availability of SARS-CoV-2 treatments or vaccines. Most of these patients were examined by the medical examiner because of death at home. The controls consisted of a contemporaneous consecutive population of 46 age and sex-matched individuals who were PCR-negative for SARS-CoV-2 (COVneg) and died during 2020. Postmortem examinations and histologic confirmation was done according to standard protocol for potentially infected patients. Samples for PCR for SARS-CoV-2 were collected at the time of postmortem examination in all cases. Full toxicology screens were done on every patient. Clinical information collected at the time of postmortem examination was blindly reviewed and catalogued, including patient demographics, time from death to postmortem examination, cause of death, clinical history as available, full toxicology results, and gross and microscopic pathologic findings where available. Lung and heart pathologic findings were coded 1–4 for types of pathologic findings. All clinical information was provided by EC and MB and reviewed and recorded by MEHH.

### Immunohistochemical analysis

Formalin-fixed paraffin-embedded serial 4-micron thick sections of left and right ventricles were examined from each case using antibodies directed against CD42 (platelets), CD15 (myeloid cells), CD68 (monocytes and macrophages), CD 56 (natural killer cells), CD34 (stem cell antigen found on endothelial cells), C4d, fibrin, and myeloperoxidase (neutrophils and neutrophil extracellular traps (NETs). Hematoxylin and Eosin (H&E) stained sections were also examined. Samples were stained as a batch (with one stain and controls) using automated platforms and standardized reagents. A table of details is available on request. The stained slides were scanned using whole slide imaging (Aperio Techologies, Vista, CA). Each slide was accessed on an Aperio web viewer. Each slide was entirely viewed at 5x, 10x, and 20x. Slides were graded using a 0–3 scale where 3 indicated the marker was present in every field at 20 × and 1 indicated the marker was present in any field at 10x. Grade 2 indicated that some fields at 20 × showed staining, but many did not. The tissue distribution of the marker was also noted. All slides were examined by a single observer (MEHH). At review, it was noted that one COVpos case was autolyzed and was not included. Excluding these, we analyzed 48 COVpos and 46 COVneg cardiac samples. Areas of left and right ventricular tissue were similarly selected from each heart.

### Statistical analysis

We used a Welch 2-sample T-test to determine significance between COVpos and COVneg samples for immunohistochemical and clinical characteristics. The test was adjusted for multiple comparisons using the False Discovery Rate method. We further explored the relationships between the markers and patients using a cluster (heatmap) analysis. This was a cluster analysis on the markers to find similarities among the expression of the markers across subjects and clusters the subjects to find similarities. The clustering was done using marker data and COVID-19 status. The results are displayed as a grid of colors indicating the actual marker scores but sorted by the results of the cluster analyses so that similar markers and similar subjects are grouped within the grid, also highlighting their COVID-19 status at death [8, 9].

## Results

Clinical characteristics are shown in Table 1.

**Table 1:**
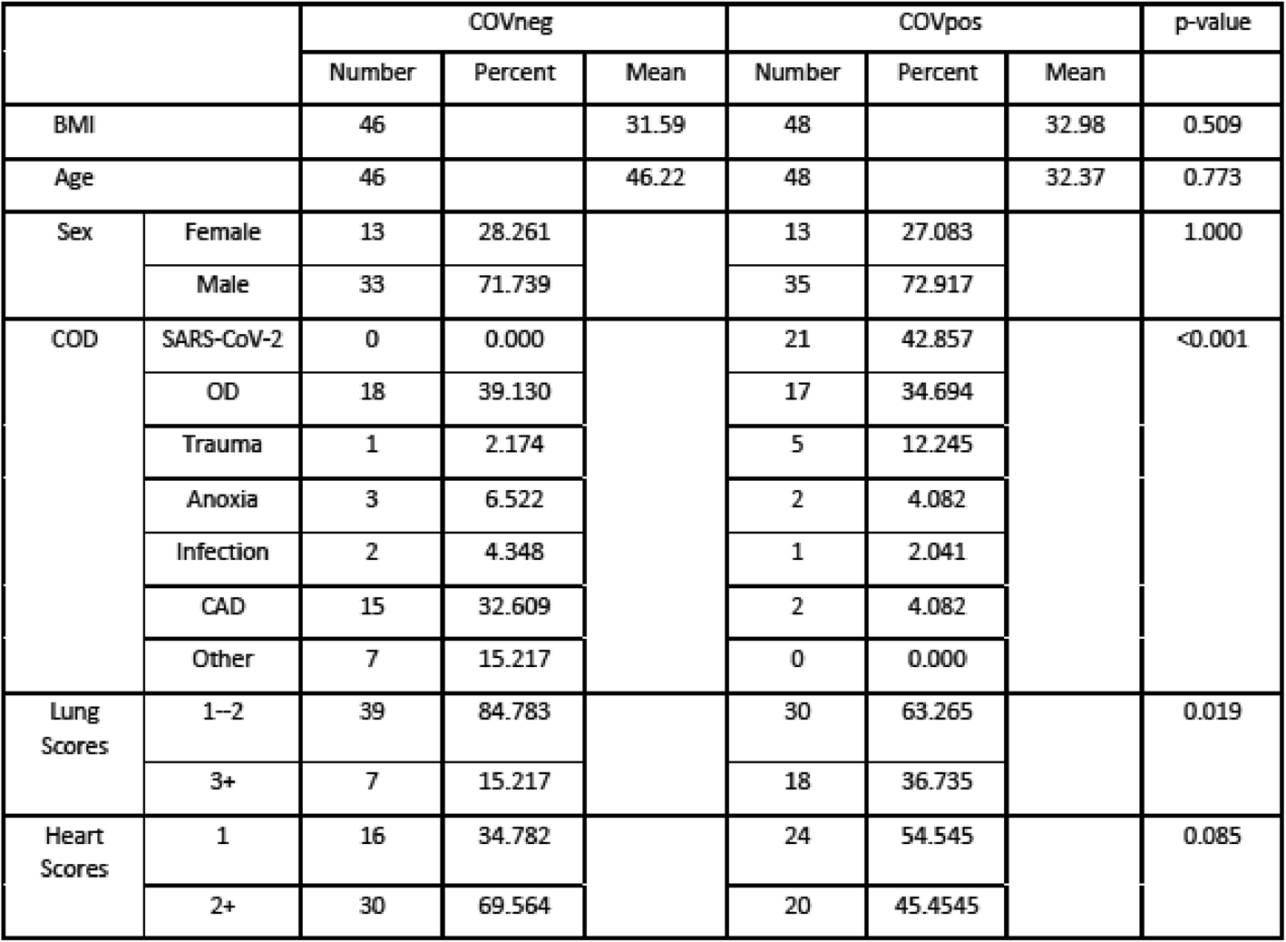
COVpos and Covneg population characteristics

COD refers to Cause of Death as recorded by the medical examiner. OD is accidental or suicidal drug overdose. CAD is coronary artery disease. Lung refers to scores for pathologic lung findings: 1 = normal lung, 2 = edema only, 3 = pneumonia, 4 = diffuse alveolar damage and 5 = vascular thrombi. Heart refers to scores of cardiac pathologic findings: 1 = normal heart, 2 = hypertensive cardiac disease, 3 = CAD, 4 = other cardiac causes including aortic stenosis, alcoholic cardiomyopathy and fatty infiltration. Cardiac and lung exams were not done on all cases

### Clinical characteristics

Age, Sex and BMI results were not different between the two groups. Average time from discovery of the deceased patient to postmortem examination was 34.7 h for the COVpos and 26.8 h for the COVneg patients. Full toxicology screens were done on every patient. Both COVpos and COVneg patients had positive toxicology in cases of drug overdose, and in patients with a history of drug abuse. Diabetes status was unknown. Cause of death (COD) was significantly different between the COVpos and COVneg groups which was primarily due to a higher rate of SARS-CoV-2 related death (COVID-19) in the COVpos patients and a higher rate of coronary artery disease (CAD) related death in the COVneg patients. Lung pathologic findings were significantly more prevalent in the COVpos patients and primarily in those who were COVpos and whose COD was SARS-CoV-2 (COVID-19) regardless of whether SARS-CoV-2 was detected prior to death or only at postmortem examination.

### Histologic features

Careful review of H&E-stained slides of each case showed no cases of lymphocytic myocarditis according to established histologic criteria [10]. No inflammation associated with individual cell myocyte necrosis was seen in any case. In some COVpos cases, small aggregates of mononuclear cells were seen, but few if any of these cells were lymphocytes. Scattered large ischemic myocardial areas were seen associated with typical inflammation in some cases, especially among the COVneg cohort who had more chronic cardiovascular disease. Fibrin aggregates were noted scattered in the myocardium of many COVpos cases (in and around capillaries, arterioles and venules). In many cases, fibrin clots were seen in arterioles and venules or within ventricular cavities. COVneg cases showed only rare fibrin aggregates and no significant intracavitary fibrin aggregates.

### CD15 (cells of myelogenous origin, monocytes): Fig. 1a

**Fig. 1:**
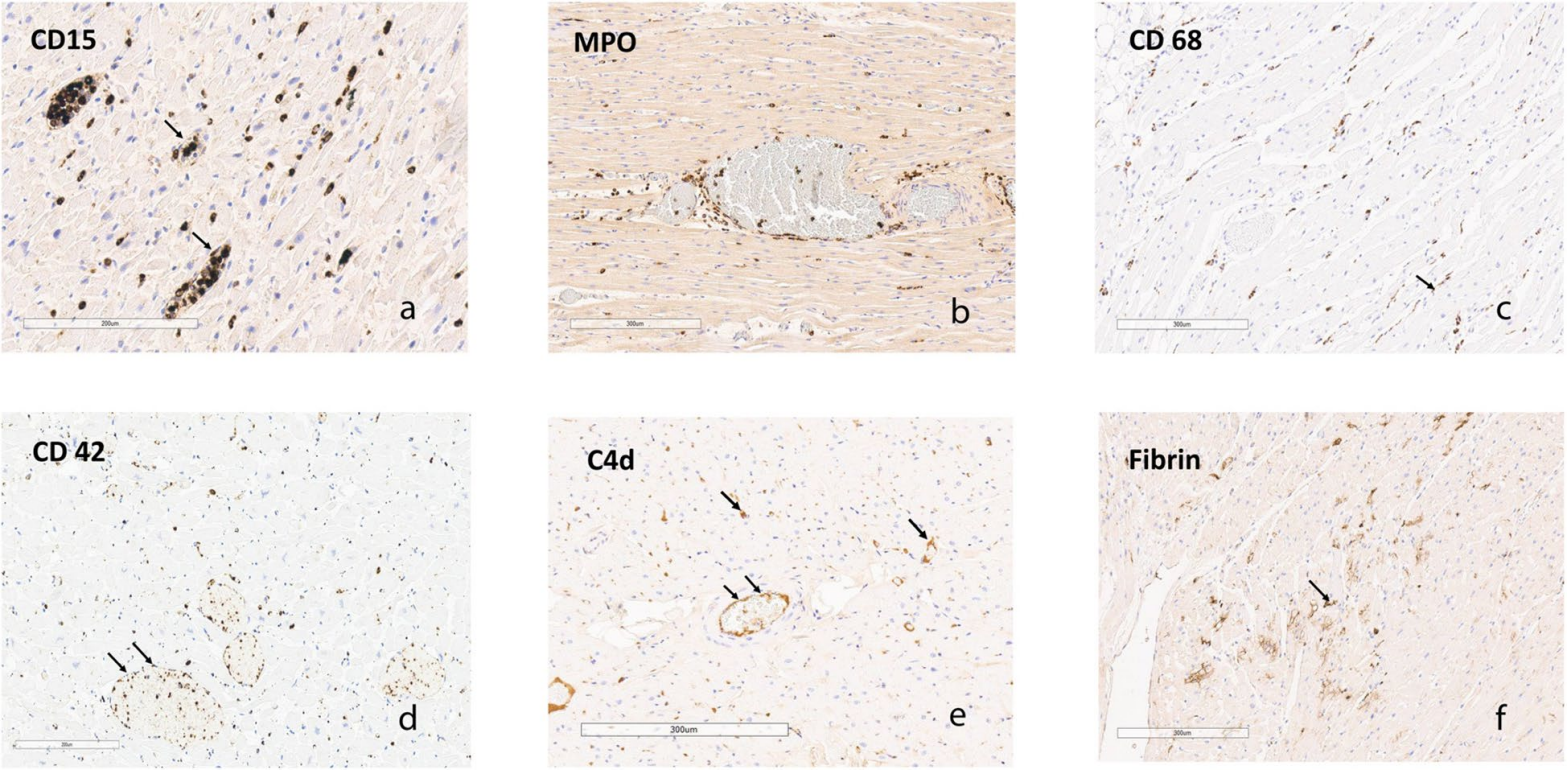
Distribution of Immunohistochemical Staining in COVpos Heart Tissue Sections. Staining of sections of heart tissue from COVpos patients with various immunohistochemical markers. The scale indicates the relative magnification of each photomicrograph. Arrow highlights capillaries; Double arrow highlights arterioles or venules. See text for explanation of the findings

Abundant CD15 was found in arterioles, venules and capillaries in many COVpos cases, often associated with interstitial cells. In some cases, CD15 positive cells lined the endothelium resembling the CD42 (platelet) distribution. Areas of myocyte ischemia in some hearts had large deposits of CD15 positive cells associated with necrotic myocytes and debris. Some similar staining was found in COVneg hearts, in areas of previous myocyte damage. Mean grade for COVpos = 1.56 ± 0.91; Mean grade for COVneg = 0.98 ± 0.97, *p* < 0.001.

### MPO: Fig. 1b

MPO staining in COVpos cases was distributed in arterioles, venules, and scattered in the interstitium like CD15 staining. Interstitial staining suggests neutrophils and NETs (8). No staining in this distribution was seen in COVneg cases. Mean grade for COVpos = 1.496 ± 1.052; Mean grade for COVneg = 0.550 ± 0.654, *p* < 0.001.

### CD 68 (macrophages, lysosomes): Fig. 1c

COVpos cases showed little morphologic evidence of macrophage activation since most stained cells were spindle shaped rather than plump. Small numbers of macrophages were found scattered through the interstitium like the CD15 staining pattern. Many cells were found in areas of myocyte damage and necrosis. Staining of COVneg cases was confined to areas of myocyte damage. Mean grade for COVpos = 1.73 ± 1.09; Mean grade for COVneg = 0.048 ± 0.215, *p* < 0.001.

### CD42 (platelets): Fig. 1d

Platelets were found mostly in venules and capillaries, but occasionally scattered within the interstitium. In some COVpos cases we observed a beading pattern of platelets along venules. Platelets were rarely found in COVneg cases. Mean grade for COVpos = 0.96 ± 0.84; Mean grade for COVneg = 0.000 ± 0.016, *p* < 0.001.

### C4d (complement component): Fig. 1e

C4d was mainly found attached to arteriolar and venular endothelium in COVpos cases. Occasional cases had focal or general capillary staining, but this was uncommon. In addition, autostaining of necrotic myocytes was observed by H&E staining including subendocardial necrosis. This autostaining was not considered to be positive in the scoring of the slides. Occasional arterioles had muscularis staining, highlighting necrosis of smooth muscle in these cases. Rare C4d staining was found in COVneg cases. Mean grade for COVpos = 0.662 ± 0.866; Mean grade for COVneg = 0.008 ± 0.052, *p* ≤ 0.001.

### Fibrin: Fig. 1f

Fibrin aggregates were found scattered in the myocardium of many COVpos cases. In addition, fibrin aggregates were found adherent to the endothelium of arterioles and venules, within arteriolar walls, and occasionally in subendocardial regions. Such staining rarely was found in COVneg cases. Mean grade for COVpos = 1.092 ± 0.895; Mean grade for COVneg = 0.108 ± 0.276, *p* < 0.001.

### CD56 (natural killer cells): not shown

CD56 was negative in all cases of both COVpos and COVneg samples. Controls run with the test cases showed appropriate positive and negative staining.

### CD 34 (stem cell vascular antigen): not shown

**Fig. 2:**
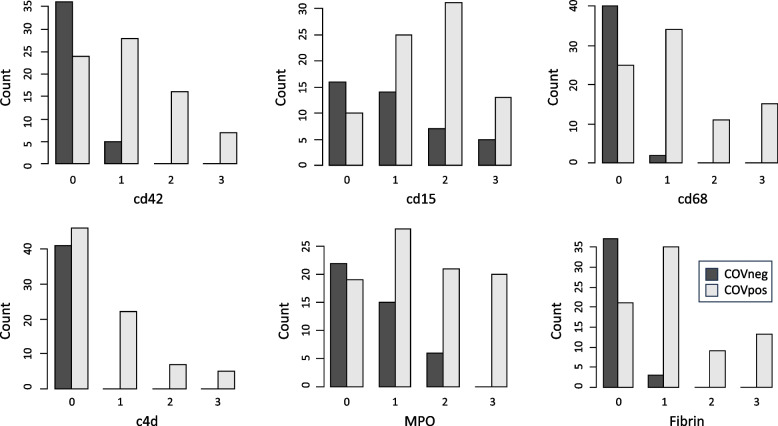
Comparison of Immunohistochemical Findings in COVpos and COVneg Heart Tissue Sections. Bar chart showing distribution of immunohistochemical staining in COVpos and COVneg heart sections. CD15 stains mature and immature myeloid cells; MPO stains neutrophils and NETs; CD 68 stains monocytes and macrophages; CD42 stains platelets; C4d stains complement component of the same name; Fibrin stains fibrin

CD 34 staining patterns in many COVpos cases resembled the typical pattern of staining observed in heart biopsy sections with smooth outlines of most uninjured capillaries [11]. This was especially true of COVneg cases. In a subset of COVpos cases, a feathering pattern of vascular staining was observed, suggestive of vascular proliferation. This vascular proliferation change was more common in COVneg subjects with obvious ischemic cardiac damage. Some cases (both COVpos and COVneg) showed a random, patchy lack of capillary staining in random areas, suggestive of capillary loss Fig. 2.

**Fig. 3:**
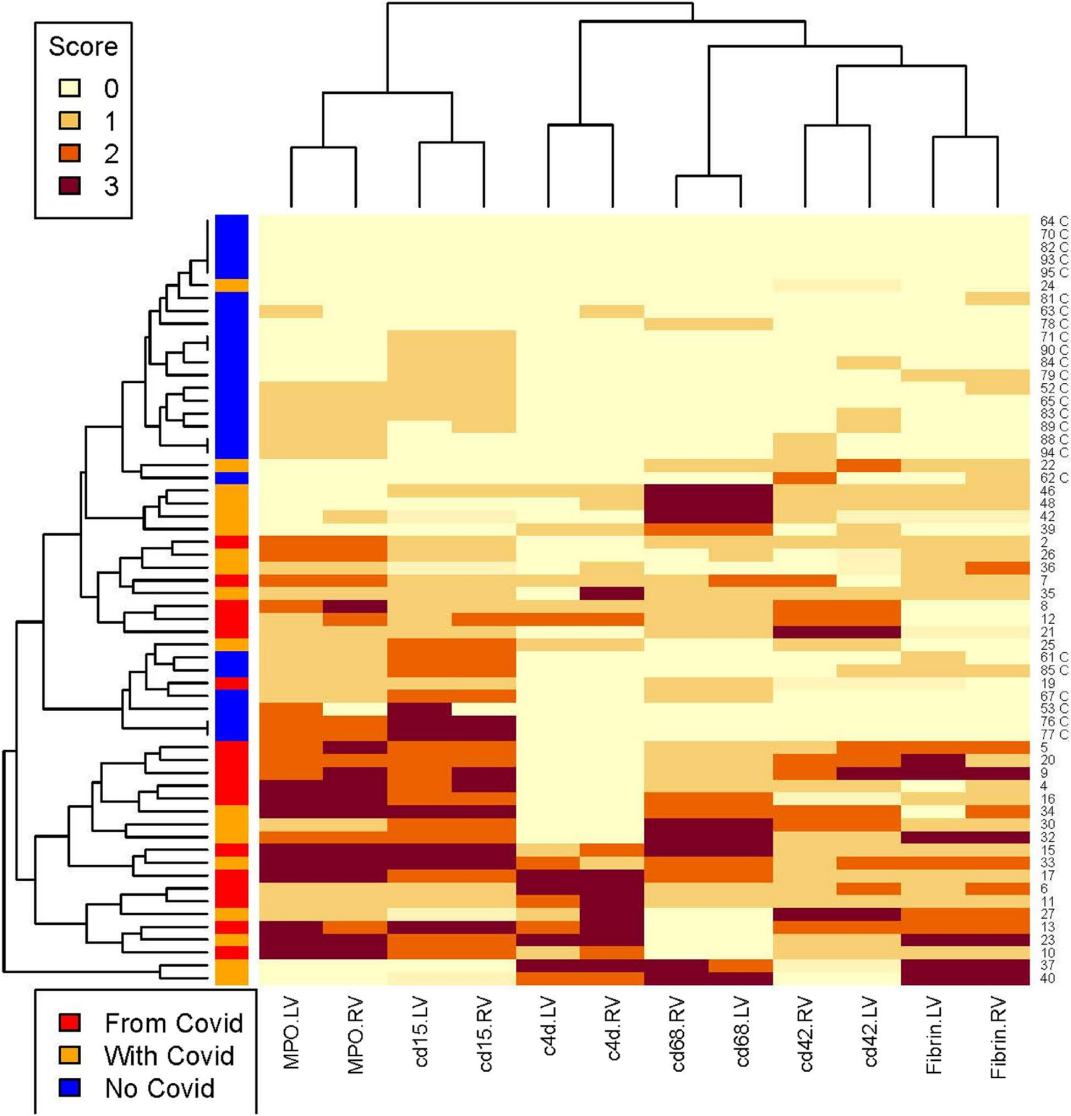
Cluster Analysis of COVpos and COVneg cases by SARS-CoV-2 status and Marker Scores. In this cluster analysis, subjects are clustered by similarity in marker scores and COVID-19 status. The large red, blue and orange bar on the y-axis refers to the COVID status: Red bar = subjects whose COD was SARS-COV-2 disease (COVID-19). Orange bar = subjects who were positive for SARS-COV-2 at postmortem examination but whose primary COD was not COVID-19. Blue bars = subjects who were negative for SARS-CoV-2 at postmortem examination. Marker names are on the X axis. Marker grades are displayed as various colors. Pale yellow = no staining (grade 0). Bright yellow = minimal staining (grade 1). Orange = moderate staining (grade 2). Brown = large amounts of staining (grade 3). LV = left ventricle; RV = right ventricle

We found a significant difference between COVpos and COVneg samples in the amount and pattern of staining for fibrin, CD42, CD68, CD15, MPO, and C4d. All differences were statistically significant (*p* < 0.001). There was no significant difference between the left and right ventricles for amount or pattern of staining in COVpos or COVneg cases (intracase variability was low). Among the COVpos cases, only 5/48(10%) had no marker staining or H&E evidence of fibrin thrombi. The mean number of positive markers in the COVpos cases was 3.70 ± 1.74 markers/case. Among the COVneg cases, 20/46 (43%) had no marker staining or H&E evidence of fibrin thrombi. The mean number of positive markers in the COVneg cases was 0.95 ± 1.09 markers/case. A cluster analysis appears as Fig. 3 to further illustrate these findings.

## Discussion

**Fig. 4:**
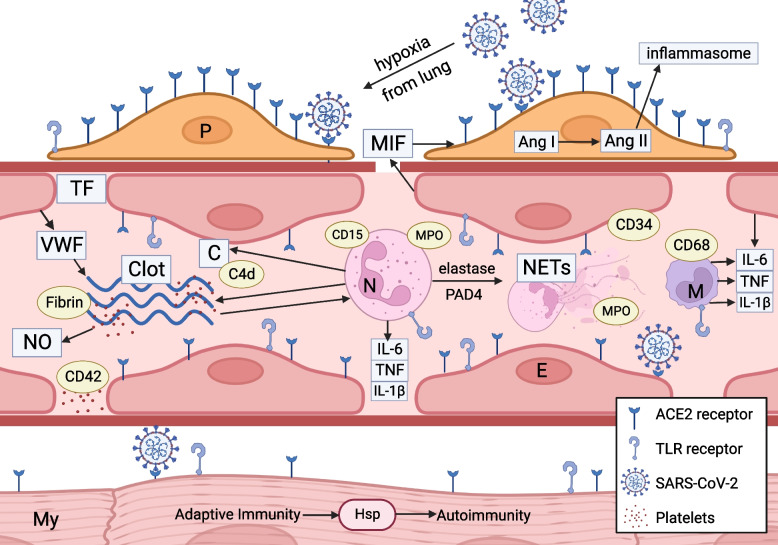
Graphical illustration of Mechanisms of Injury in COVpos Heart Tissue Sections Simplified graphical illustration of mechanisms of injury by SARS-CoV-2 in the heart. Graphic displays the mediators and cells which play a role in this process which predominantly involves cardiac vasculature. Lung infection with SARS-CoV-2 leads to hypoxia and activation of inflammatory cytokine elaboration in the blood of infected patients. Pericytes (P) have the highest concentration of ACE2 receptors in the heart. SARS-CoV-2 can thereby easily infect pericytes. ACE2 receptor downregulation causing accumulation of Angiotensin II promotes generation of cytokines (IL 6, IL 1b, TNF) accelerating inflammation. Toll Receptor (TLR) binding to ligands generated by infection activates intracellular signaling cascades which lead to inflammatory cytokine generation. Activation of endothelial cells (E) generates inflammatory cytokines as well as macrophage inhibitory factor (MIF) that leads to gaps in the vascular basement membrane promoting vascular permeability and extravasation of neutrophils, NETS, platelets, and Fibrin into the interstitium. Neutrophil (N) activation and neutrophil extracellular trap (NETS) generation (through the action of elastase and Peptidylarginine deiminase 4(PAD4) play a pivotal role. They stimulate coagulation and complement activation, creating positive feedback loops between endothelial cells and platelets. Platelets generate nitrous oxide (NO), another potent mediator. Endothelial cells elaborate von Willebrand factor (VWF) which is an important accelerator of coagulation. Cell necrosis caused by the autoinflammatory process exposes cellular and viral antigens that initiate adaptive immunity as well as autoimmunity against myocyte and endothelial antigens with homology to SARS-CoV-2 antigens such as heat shock protein (HSP). Myocyte injury can be initiated by direct infection, ischemia generated by the vascular injury and hypoxia, and by autoimmunity against myocyte antigens. Yellow labels indicate markers used in the study whose roles are highlighted in the diagram

The overall pattern of immune factor staining in most of the COVpos cases supports the hypothesis that innate immune activation (autoinflammation) is a major mechanism for cardiac injury due to SARS-CoV-2 infection. The graphic in Fig. 4 depicts the interaction of various autoinflammatory pathways.

In severe infection with SARS-CoV-2, the virus is likely disseminated to other organs via infected monocytes and circulating vesicles from infected cells [6]. Because many ACE2 receptors are found in the heart, particularly on pericytes, the vasculature of the heart is a convenient target for viral proliferation and innate and adaptive immune system activation [6, 12–15]. Because of SARS-CoV-2’s molecular mimicry of humans’ proteins which have analogous peptide sequences, immune responses against SARS-CoV-2 can be directed towards human proteins. In particular, molecular mimicry was revealed between the human SARS-CoV-2 and heat shock proteins (Hsp), which has been linked to Guillain-Barré syndrome and other autoimmune illnesses. The autoimmune response of SARS-CoV-2 to Hsp further damages the vascular endothelial lining throughout target organs like the heart, leading to activation of vascular endothelium with subsequent damage, coagulation, complement activation, and leukocyte infiltration [12–15].

Our study highlights the pivotal role of neutrophils in cardiac injury caused by SARS-CoV-2 infection. The increased presence of neutrophils and MPO in cells as well as in interstitial staining debris is evidence of NET formation. Other features include fibrin aggregates, platelets, monocytes, and complement (C4d), which were most prominent in or around venules and arterioles. This combination of factors was not seen in COVneg cases, suggesting this type of inflammation is unique to those infected with SARS-CoV-2. COVneg subjects had more chronic cardiac disease which was often the primary COD. Despite this reality, autoinflammatory markers were not seen in the COVneg heart samples.

Recent studies on the lung, serum, and hearts of patients hospitalized for SARS-CoV-2 disease have highlighted the pivotal role of neutrophils [16–18]. Neutrophils, together with mononuclear cells, are the first cells attracted to the SARS-CoV-2-infected alveoli recruited by interferons, CCL2, IL-6, IL-1, and other cytokines. On site, they eradicate the virus-infected cells, produce proinflammatory mediators, and secrete various proteases (via NETosis or independently on NETs). One report provides evidence of massive degranulation of neutrophils in the serum [17]. Our study supports these findings as degranulation was found in the cardiac tissue, manifested by MPO positive debris in the interstitium [16]. The majority of COVpos cases did not appear to have chronic myocardial injury upon histologic examination. Most cases had scattered areas of ischemic myocyte damage, confirmed by staining with C4d. Often this ischemic damage was localized to the subendocardial space [16–18]. This is a postmortem study of patients dying in suspicious circumstances requiring postmortem examination by the medical examiner. Little information about symptoms prior to death was available. We thus cannot predict which of these patients, whether COVpos or COVneg would have experienced clinical cardiac symptoms had they lived longer. 

Many autopsy studies have been published since the SARS-CoV-2 pandemic started and illustrate a variety of pathologic features. A recent meta-analysis of 50 studies comprising 548 autopsy cases found that myocyte necrosis and edema were the most common cardiac findings (median prevalence 100% for necrosis; 55.5% for edema) in patients dying of SARS-CoV-2 infection [19]. The analysis also showed evidence that SARS-CoV-2 can be found in the heart in cardiomyocytes and endothelial cells. The median prevalence of virus in these studies was 60.8%, although the amount of virus found in any study using immunohistochemistry or in situ hybridization was minimal. Direct viral infection of the heart may act to further trigger the autoinflammation that is prevalent in these cases. Further work is needed to understand the relationship between direct infection and autoinflammation. In our cohort, we did not do staining to detect viral antigens.

Importantly, we saw no evidence of lymphocytic myocarditis in any COVpos or COVneg cases. The meta-analysis of cardiac findings in patients dying of SARS-CoV-2 infection emphasized the confusion associated with the diagnosis of myocarditis. In the meta-analysis, no convincing evidence of lymphocytic myocarditis was found that could not be explained by the improved sampling that autopsy tissue permits relative to endomyocardial biopsy. The median prevalence of myocarditis was 0.0%. Our data support this conclusion. We saw no evidence of lymphocytic myocarditis in our study cases and all the inflammatory cells seen stained as monocytes, macrophages, or neutrophils [19].

Some confusion about myocarditis in SARS-CoV-2 infection has been generated by the adoption of cardiovascular magnetic resonance (CMR) imaging criteria for myocarditis rather than histologic criteria [20]. CMR findings include T1 mapping abnormalities (suggesting diffuse myocardial changes such as diffuse fibrosis and/or edema); T2, short tau inversion recovery, or T2 mapping abnormalities (more specific for myocardial inflammation, as occurs in acute myocarditis); late gadolinium enhancement (LGE, suggestive of acute myocardial injury and/or myocardial fibrosis); or pericardial involvement—all of which can indicate cardiac pathologies associated with SARS-CoV-2 infection [4–6]. None of these CMR findings are specific evidence of lymphocytic myocarditis according to the classic Dallas criteria [10]. All could be related to the findings we report in which there is myocardial damage, ischemia, edema, fibrin thrombi, and cellular infiltrates of neutrophils, monocytes, and platelets. Our findings lend credence to the conclusions of several authors that CMR findings are more likely related to myocardial injury and edema than lymphocytic infiltration with associated myocyte damage [4–6]. We saw no evidence of lymphocytic myocarditis. By contrast, we found immunohistochemical evidence of coagulopathy, neutrophil infiltration, complement activation, and vascular injury. These features were seen only in COVpos samples and could be responsible for the imaging patterns detected in such cases.

### Limitations

This is a retrospective, single institution study of a selected population. We selected markers based on our hypothesis that autoinflammation was the most important cause of potential or actual cardiac damage. We did not investigate antibody-mediated responses or search directly for viral antigens. Those other mechanisms could be investigated in a further study.

## Conclusion

Our study provides morphologic evidence that systemic innate immune activation (autoinflammation) in the cardiovascular system is a major mechanism of cardiac injury in medical examiner cases dying with SARS-CoV-2 infection compared to cases negative for this virus with comparable demographics and causes of death. The cases were collected prior to the availability of treatments or vaccines for SARS CoV-2 infection which could alter pathologic features of cardiac involvement. We cannot predict whether patients with these findings would have experienced clinical cardiac disease if they had lived longer.

## Data Availability

The datasets used and/or analyzed during the current study are available from the corresponding author on reasonable request.

## Abbreviations

*SARS-CoV-2*: Severe Acute Respiratory Syndrome Coronavirus-2
*H&E*: Hematoxylin and Eosin
*CD*: Cluster designation
*NETS*: Neutrophil Extracellular Traps
*MPO*: Myeloperoxidase
*NK*: Natural Killer Cells
*NLRP3*: NLR family pyrin domain containing 3
*NFkB*: Nuclear factor kappa B
*IL-6*: Interleukin 6
*hsCRP*: High sensitivity C reactive protein
*IL-1*: Interleukin 1
*MIF*: Macrophage inhibitory factor
*TNF*: Tumor necrosis factor
*PAD4*: Peptidylarginine deiminase 4
*Hsp*: Heat Shock Protein
*CCL-2*: Chemokine ligand-2
*CMR*: Cardiovascular Magnetic Resonance
